# Primary Health Care Nurses’ Experiences of Consultations With Internet-Informed Patients: Qualitative Study

**DOI:** 10.2196/14194

**Published:** 2019-07-30

**Authors:** Anna E Sjöström, Åsa Hörnsten, Senada Hajdarevic, Agneta Emmoth, Ulf Isaksson

**Affiliations:** 1 Department of Nursing Umeå University Umeå Sweden; 2 Arctic Research Centre Umeå University Umeå Sweden

**Keywords:** eHealth, Internet, consumer health information, health literacy, patient-centered care, qualitative research, primary health care

## Abstract

**Background:**

Most people in modern societies now use the Internet to obtain health-related information. By giving patients knowledge, digital health information is considered to increase patient involvement and patient-centered interactions in health care. However, concerns are raised about the varying quality of health-related websites and low health literacy in the population. There is a gap in the current knowledge of nurses’ experiences with Internet-informed patients.

**Objective:**

The objective of this study was to explore primary health care nurses’ experiences of consultations with patients who present health-related information from the Internet.

**Methods:**

This is a qualitative study based on interviews with 9 primary health care nurses. Data were analyzed using qualitative content analysis. Results are reported according to the consolidated criteria for reporting qualitative research guidelines.

**Results:**

The phenomenon of Internet-informed patients was considered to change the usual rules in health care, affecting attributes and actions of patients, patterns of interactions in consultations, and roles of nurses and patients. Three categories were identified: (1) Facing the downsides of Googling, (2) Patients as main actors, and (3) Nurse role challenged. Although the benefits of health-related Internet information were described, its negative consequences were emphasized overall. The problems were mainly ascribed to inaccurate Internet information and patients’ inability to effectively manage the information.

**Conclusions:**

Our study suggests ambivalent attitudes among nurses toward health-related Internet information. In order to promote equitable care in the digital era, increased awareness in health care about useful strategies for overcoming the difficulties and embracing the benefits of conferring with Internet-informed patients seems to be a legitimate goal.

## Introduction

In the ongoing digitalization of modern societies, health care has also been affected. The widely used term *eHealth* refers to the use of information and communications technology (ICT) in health care. The World Health Organization has acknowledged the potential of ICT to play a major role in improving public health and it urges member states to prioritize the development of eHealth technology [1]. The vision of the Swedish government is that by 2025, Sweden will be the best country in the world at using eHealth to make it easier for people to achieve good and equal health and to increase patients’ participation [2]. Digital services currently delivered by Swedish health care include electronic health records, electronic prescriptions, and online communications with health care professionals (HCPs) [3]. To provide health-related Internet information (HRII) as a part of eHealth, the Swedish county councils and regions provide its citizens with a national website, 1177, that contains health-related information and advice that is quality assured by medical experts [4]. However, nearly 80% of the population choose Google and only 8% use the national website to search for information about medical conditions [5]. Google, or similar search engines, is also the primary choice, internationally, for people who search for HRII [6,7].

About 85% of the Swedish population use the Internet to various degrees to obtain health-related information [8] and studies show similar numbers in the rest of Europe and the United States [6,7]. Patients’ reasons for their HRII searches include learning about a medical condition, deciding whether a health care visit is needed, preparing for a consultation, or validating information received during a health care consultation [9,10].

HRII has been described as accelerating a shift among patients, from passive recipients to active consumers of health information [11], and among patient-HCP relationships, from hierarchical to more reciprocal [12]. By equipping patients with knowledge about health, HRII may increase their involvement in care and ability to make informed decisions, which could increase person-centered interactions between patients and HCPs [11,13-15]. The World Health Organization and policy makers across the world have emphasized a need for health care to become more person-centered [16-18].

However, concerns have been raised about the general quality of HRII; several studies have shown significant variation in the accuracy and readability of HRII and only a minority of health-related websites has been assessed as offering high-quality information [19-21]. Concerns also include the fact that a disquieting proportion of European and US populations have limited health literacy skills [22,23], which means they are not likely able to accurately interpret and use relevant health information and resources to achieve their health goals [24]. The consequences of inaccurate HRII and patients’ various abilities to evaluate that information include increased worries, inadequate self-care, postponements of clinical encounters, and tensions in the patient-HCP relationship [25,26]. The ability to manage health knowledge acquired from digital sources (ie, eHealth literacy) requires additional skills, including the appropriate use of digital tools [27].

Previous research on HCPs’ views of Internet-informed patients has mainly focused on physicians, showing mostly positive or neutral attitudes [28,29] but some negative attitudes as well [25]. Previous studies exploring nurses’ views of patients that bring up HRII in clinical encounters have used quantitative approaches and reported generally positive attitudes [30,31].

The dedication of HCPs seems a fundamental prerequisite to the success of policy makers’ and health care providers’ efforts toward eHealth. The attitudes of HCPs toward HRII and increased patient involvement are also central to person-centered care. However, there is a lack of research on nurses’ views of their consultations with Internet-informed patients. Swedish primary health care nurses (PHNs), who most often work independently of general practitioners, usually represent the first contact for patients in need of care or health advice [32]. Central work tasks of Swedish PHNs include counseling patients over the telephone, guiding them to the right HCP or level in the health system, preventing illness, and treating patients in their catchment areas [33]. The aim of this study was, therefore, to explore PHNs’ experiences of consulting with Internet-informed patients.

## Methods

### Study Design

A qualitative inductive approach was used. According to the Enhancing the QUAlity and Transparency Of health Research (EQUATOR) guidelines, the consolidated criteria for reporting qualitative research checklist for qualitative studies was followed during the study process [34].

### Participants and Setting

A total of 9 PHNs working at three primary health care centers participated in the study. Two of the health care centers were in a midsized city in Northern Sweden and one was in a rural area in Northern Sweden. Head managers of the health care centers were asked to assist in recruiting participants, preferably varying in age and years of working experience, for an interview about eHealth. PHNs were informed in person or at staff meetings and 9 PHNs volunteered to participate. Inclusion criteria were being a registered nurse with a specialist graduate nursing diploma in primary health care nursing. The 9 participating PHNs ranged in age from 27 to 64 years (median 42) with work experience of 4-39 years (median 18) as registered nurses and 1-23 years (median 5) as PHNs.

### Data Collection

Semistructured interviews were conducted by two of the authors (AES and AE) in 2016. An interview guide consisted of open-ended questions about the nurses’ experiences of meeting patients who present HRII during consultations. Questions from the guide included “How do you, in your everyday work, perceive patients searching for HRII?”; “What problems do you perceive?”; “What benefits do you perceive?”; and “How do patients’ presentations of HRII affect you in your everyday work?” With the interview guide as a skeleton, follow-up questions were asked and participants were encouraged to give several examples of their experiences. The recorded interviews, which were rich in data, lasted from 30 to 60 minutes (median 50) and were transcribed verbatim.

### Data Analysis

The interviews were analyzed using qualitative content analysis as described by Graneheim and Lundman [35]. Initially, the data were read thoroughly to acquire a basic understanding of the content. Text that corresponded to the aim was divided into meaning units and condensed (ie, shortened, but with the core message kept). The condensed meaning units were interpreted and labeled with codes. All authors discussed the codes and, based on similarities and differences, three categories and nine subcategories were identified. The authors continuously discussed the coding and categorization until consensus was achieved, which strengthens the study’s trustworthiness [36].

### Ethical Considerations

The study was conducted according to the ethical principles described in the Helsinki declaration [37]. Participants were provided with information about the study, both verbally and in a written letter. Participation in the study was voluntary, and the participants were informed that they could withdraw at any time without stating any reason. Informed consent was obtained from both participants and managers of the primary health care centers. Data material and personal information have been handled confidentially. The Regional Ethical Review Board at Umeå University approved the study (approval number: 2014-179-31M).

## Results

### Overview

The PHNs unanimously described consultations with Internet-informed patients as common in their everyday work. Internet-informed patients were described to be of all ages, although mainly of working age. The analysis of the interviews resulted in three categories: (1) Facing the downsides of *Googling*, (2) Patients as main actors, and (3) Nurse role challenged. Each category consisted of three subcategories. The theme that emerged and encapsulated the essence of experiences related by the PHNs was *Internet-informed patients change the rules of the game* (see Table 1). Each subcategory in the following sections is illustrated with a quotation.

**Table 1:** Categories and subcategories of the theme, *Internet-informed patients change the rules of the game*, emergent in the analysis.

Category	Subcategories
Facing the downsides of *Googling*	Confusion due to contradictions; Disputes related to differing opinions; Unfounded anxiety among patients
Patients as main actors	Patients as lay experts; Self-care initiatives facilitated; Patients as equal partners
Nurse role challenged	Being considered unnecessary; Importance of keeping up-to-date; Coaching instead of controlling

### Internet-Informed Patients Change the Rules of the Game

The overarching theme, *Internet-informed patients change the rules of the game*, summarizes the interpreted essence of the PHNs’ experiences of the considerable influence of HRII and knowledgeable patients on health care consultations. This influence was described to generate changes in patients’ behaviors, patterns of interaction during consultations, and the roles of patients and PHNs. The overall experiences and attitudes toward these changes were marked by ambivalence, although the problems were made very explicit.

### Facing the Downsides of Googling

#### Overview

Inadequate and contradictive HRII, often as a result of patients’ free Googling for symptoms and illness, was described to frequently generate confusion, anxiety, and conflict. These downsides were described not only as unfavorable for patients, but also as challenging and time-consuming for the PHNs in their everyday work.

#### Confusion Due to Contradictions

The nurses reported that during consultations, patients often expressed confusion and uncertainty about what information to trust, due to the contradictory nature of information on different websites. Inaccurate HRII and search methods were emphasized as important factors in confusing the patients. Free Googling was highlighted as a significantly critical activity, since many patients were described as unable to distinguish reliable from unreliable HRII when sorting through search results.

People tend to over-seek for symptoms and illness on the Internet. They use Google and end up on the wrong websites. Because it is not like the most credible websites will be on top of the search results list. I wish they could go directly to 1177. They would then receive the correct information at once, without having to get so confused...

Parents of toddlers stood out to the nurses as a group particularly inclined to becoming confused by HRII. This was ascribed mainly to their frequent visits to different parenting forums and groups, where the false belief flourished that parents’ experiences around their children were automatically transferable to other parents’ children. Inaccurate methods of obtaining and critically analyzing HRII were described as occasionally causing parents to reject important recommendations, such as the necessity of immunizations.

#### Disputes Related to Differing Opinions

The PHNs described patients’ presentations of HRII to be a frequent generator of conflicts. Such disagreements arose when patients presented HRII or interpretations of HRII that went against the PHNs’ knowledge or, from a professional point of view, were even completely false. In cases of differing opinions about, for example, diagnoses or desired treatments, patients with firm ideas were described as challenging, frustrating, and stressful. The nurses considered themselves obliged to respect the patient’s opinion but also to be clear about their view on the matter and to refer patients to reliable websites. The nurses emphasized that mutual and respectful dialogue was essential for patients to be satisfied with their care despite conflicting opinions.

A recurrent conflict of opinions occurred when the PHN advised self-care or a change of lifestyle as a primary treatment strategy to patients who preferred *quick-fix* options they had read about online.

When there are several steps to treat a disease, well, then they immediately wish to go to step three. Instead of trying the first two steps. When they have an eye infection, we can say, “Wash your eyes for a week and come back if it doesn’t get better.” Well, then they instead want you to prescribe antibiotics at once so they can go to work or send their kids to school...It seems tough to endure.

#### Unfounded Anxiety Among Patients

Unfounded anxiety among patients was unanimously experienced as a common consequence of HRII. Due to online searches, many patients were reported to interpret minor symptoms as the “worst imaginable disease,” not infrequently cancer. Searching indiscriminately for HRII was felt to be particularly destructive for people who already suffered from health anxiety, since it was regarded to increase anxiety considerably.

...or when they call about a headache. Well, then they imagine it is a brain tumour. Since they read on the Internet that for some people it started with a headache, well, then it has to be a brain tumour...

A group described as particularly disposed to become anxious from obtaining HRII was parents of toddlers. Their anxiety was mainly ascribed to unreliable websites and forums where they read other parents’ shared experiences of severe symptoms and diseases and interpreted their own child’s banal symptom to signal a life-threatening disease. The PHNs described frequently being contacted by anxious patients and parents, who after obtaining HRII wanted to be reassured and to rule out dangerous conditions that could purportedly be causing their symptoms. Dealing with patients or parents who were anxious after obtaining HRII was often perceived as problematic, since these patients tended to seem obstinate, take up more time, and show more dissatisfaction with health care than other patients.

### Patients as Main Actors

#### Overview

Knowledge acquisition through HRII was considered to enable patients to adopt a more active and confident role in their care. Benefits of such an active patient role included the patients’ increased ability to manage self-care activities at home, which was considered to relieve the health care system. However, the PHNs also described the increased patient involvement as partly problematic, since many patients were perceived to adopt a lay expert role that they lacked the competence to manage.

#### Patients as Lay Experts

The PHNs described how easily accessible HRII made several patients seem to consider themselves medical experts. These patients were characterized by their use of advanced medical terminology and clear conceptions of diagnoses and treatment requirements. Patients who were perceived as self-declared experts and who had already formed their opinion prior to contact were reported to be generally less receptive than others to information given by the PHNs and more likely to question the competence and knowledge of the PHNs.

The perceived development of ever more *lay experts* was described as problematic, since many of these patients were considered to have difficulty properly interpreting the HRII they found. These problems were explained by a lack of medical experience among patients, an attribute that was considered important in making certain medical decisions.

Most often, patients start by saying what diagnosis they believe they have, instead of saying, “Hi! I would like counselling. I have these problems...” That almost never happens, but rather they [claim to] have a clear diagnosis.

#### Self-Care Initiatives Facilitated

The PHNs stated that the Internet was a useful source of health information, but only if the HRII was obtained from reliable websites. Reliable websites were described as scientific or governmental sources providing evidence-based information. These reliable websites were considered to play an important role in giving patients advice on self-care activities. The Swedish national website, 1177, was highlighted as a particularly valuable resource, since it allowed patients to assess whether they needed professional help right away or if they could try self-care as the first step of treatment. Since advice on self-care is a central part of the PHNs’ everyday work, they regarded reliable HRII from sources such as 1177 to facilitate their work and thereby reduce their workload.

All the nurses stated that they frequently referred patients to the 1177 website for advice on specific medical conditions and self-care. This website was considered to promote safe and equal care, as the self-care advice was based on evidence and not on a specific carer’s personal experience.

The benefit [of HRII] is that patients sometimes can avoid seeking healthcare. That must be said to be the absolute benefit. That a lot can be handled at home.

#### Patients as Equal Partners

Nurses with many years of experience in the profession reported that patients are more involved in their own care today than they were 15 or 20 years ago. The increased involvement was believed to be linked to, and supported by, patients’ increased knowledge about health, which was ascribed to their use of the Internet.

The patients were generally described as well-prepared prior to meetings in person or over the telephone, which was considered favorable for both patients and PHNs, but only if patients had accessed accurate HRII. Well-prepared patients were considered better equipped to ask relevant questions and to contribute to more well-structured conversations. When patients were prepared with adequate information prior to a consultation, the nurses felt able to move quickly past basic information toward mutual reasoning about the patient’s specific problem. The ability of patients to visit reliable websites to have a consultation to control the correctness of the information accessed was mentioned as a beneficial aspect of HRII. The development of more competent, involved, and well-prepared patients was considered to contribute to an approach in which the patient and the nurses were equal partners.

I also have the opinion that we must work together with the patients to find the diagnosis, as they are the experts on themselves and how they feel. And we are able to handle the part with...well, the function of the human body and diseases and stuff...And well...then we together can figure out what it can be about.

### Nurse Role Challenged

#### Overview

The PHNs described that increasingly active and knowledgeable patients, enabled by HRII, had brought a development in which their professional role was challenged and somewhat changed. Challenges of this role change included managing feelings of being marginalized and questioned, adapting to a more coaching role, and keeping updated with the latest evidence-based knowledge.

#### Being Considered Unnecessary

Consultations with patients who had a clear perception of a diagnosis and treatment from HRII often generated feelings in the PHNs of marginalization and being considered unnecessary. Many patients were perceived to want the consultation only to have their medicines prescribed or be referred to another HCP. In these situations, the nurses felt their advice was unwanted and that the patients considered a conversation needless. Thus, the traditional gatekeeper PHN role of assessing patients’ symptoms and deciding on proper treatment or guidance was considered endangered and somewhat diminished.

It’s like...they are focused on only one solution, there are no alternatives. “I must meet a doctor! Because I have this and this.” And “I have tonsillitis, I’m sure!” Okay...And then you ask some questions and take a look at them. So...they are so sure they would rather not meet a nurse, because they just want medicine and to see a doctor.

#### Keeping Updated Is Essential

The progress of easily accessible HRII and increasingly knowledgeable patients was described to place higher demands on the professional skills of the nurses. Being well-prepared with the latest evidence-based facts and ensuring patients were provided with correct information were considered crucial to conducting credible consultations. Higher knowledge demands were considered beneficial to the quality of the PHNs’ professional role as well as for health care in general. The nurses highlighted the importance of continually visiting and keeping updated on the information on the websites that they recommended to their patients to visit for patient facts and self-care advice.

Patients were described to frequently present HRII on topics that the nurses had less knowledge about. These situations were stressful, since lacking certain knowledge was associated with feeling unprofessional. However, the nurses emphasized that being credible included being able to admit a lack of knowledge about a specific topic.

The patients having more knowledge requires more of me as a nurse. I often have to motivate and argue [with them] for them to buy what I say. Before, it was more like I could say, “this is the way it is,” and they immediately bought it.

#### Coaching Instead of Controlling

The shift in role from compliant and passive patient to more expert and active participants in care was described as having necessitated a complementary change in the role and approach of the PHN. With the massive supply of HRII, it was felt necessary for PHNs to accept reduced control over their patients. A cooperative approach, in which patients are considered the experts on their own well-being and the HCPs contribute the medical perspective, was considered optimal, although challenging. Coaching skills and respectful communications were highlighted as important in helping patients to obtain reliable information from the Internet and thereby take an active role in their own care.

Supporting patients who had become anxious or confused from searching for HRII was also described as a central and important part of the everyday work of PHNs. The significance of making a reliable impression and promoting positive consultations with such patients was emphasized. Strategies used by the PHNs in these situations included calming, supporting, arguing, normalizing, informing, and dedramatizing. Supporting patients in their evaluation of HRII, which could mean either confirming or denying the veracity of the information and guiding patients to credible websites, was considered to be a main function of PHNs in the current digital era.

One has to be very clear and informative. It is about supporting, calming, arguing...I have to argue and motivate them to understand, “you don’t have to worry, I do not suspect any serious disease.” I have to normalise [their situation].

## Discussion

### Principal Findings

The PHNs in our study thought that Internet-informed patients had changed the usual rules of health care. These changes were seen in patient attributes, patterns of interaction during consultations, and the roles of patients and PHNs. The benefits of HRII were described, but its negative consequences and difficulties were emphasized overall. Our results, therefore, show more negative attitudes among nurses to the phenomenon of Internet-informed patients than previous research with the same focus [30,31].This is in line with a study by Öberg et al, who found ambivalent perceptions among PHNs of the digitalization of health care [38].

The PHNs in our study distinguished clearly between different qualities of HRII, considering reliable HRII favorable and unreliable HRII unfavorable. Previous studies have shown that the accuracy and quality of patient-presented HRII is relevant to HCPs’ attitudes [28,30]. Like HCPs in previous studies [12,29], the PHNs in our study considered evidence-based HRII beneficial for its contribution to well-prepared patients and, therefore, more structured and fruitful consultations.

One finding in our study was that having HRII, patients challenged the nurse’s role. HRII has been described as transforming patients from passive recipients to active consumers of health information [11] and the patient-HCP relationship to more of a partnership [12,29]. This transformation is in line with the efforts of policy makers and health care systems toward increasingly person-centered care [17]. The PHNs in our study were unanimously positive toward increased patient involvement and the person-centered approach, although there seemed to be a fine line between patients being moderately involved and being overly involved. Humble expressions of involvement were described as favorable for both patients and health care systems; however, expressions of *lay expertise* and overinvolvement coupled with a lack of medical competence were perceived as problematic. Disagreements arising from patients’ presentations of false or inaccurately interpreted HRII were highlighted as challenging by the PHNs of this study, as similarly described in previous research [29,39,40]. The PHNs in our study emphasized the importance of supporting positive encounters by showing respect for patients’ opinions. This approach is in line with previous research, which suggests the importance both of acknowledging patients’ efforts to acquire knowledge and of clearly explaining the reason for divergent opinions in order to give patients a positive experience and increase the chances of patients accepting the HCPs’ advice [41,42].

The Internet has been described as a catalyst for shifting power in HCP-patient interactions, which can be regarded as a threat toward HCPs’ medical authority [11,43]. HCPs’ negative feelings about conferring with patients equipped with HRII knowledge, previously reserved for HCPs, have been attributed to perceptions of being undervalued, perceived as incompetent, or losing control of the consultation [44]. Similarly, the PHNs in our study described the lay expert role of Internet-informed patients as challenging, as it caused them to feel that their professional knowledge was being questioned, which was especially stressful when they lacked full knowledge of a specific topic. This confirms a study by Öberg et al, in which PHNs also described negative feelings about being questioned and feeling insecure when meeting well-informed patients [38]. The PHNs in our study emphasized the importance of admitting when they lacked knowledge, an attribute of HCPs that Internet-informed patients value and consider to strengthen the patient-HCP relationship [12]. HCPs who act in patriarchal ways and are unwilling to admit a lack of knowledge, on the other hand, have been shown to negatively affect patient experiences [41].

The PHNs in our study were also concerned about HRII as a potential source of negative effects on patients. Free Googling, a well-documented and dominant first strategy for patients who search for HRII [26,45,46], was emphasized as a common generator of confusion and anxiety among patients. Symptom inquiries in search engines like Google often return high rates of statistically unlikely results (eg, a headache signaling a brain tumor) [47], according to the nurses in our study, and caused notable unfounded patient anxiety. Increased anxiety after searching for HRII, especially in health-anxious people, has been reported in several studies as *cyberchondria* [48-50]. HCPs have been advised to either counsel health-anxious patients to avoid HRII [48,51] or to actively guide them to credible websites [47,52]. The PHNs’ perception that parents of toddlers are high consumers of HRII, particularly inclined to insecurity and anxiety, is in line with a study by van der Gugten et al [53]. The PHNs’ concerns about the negative effects of HRII searches on patients are confirmed by research and appear legitimate.

The PHNs suggested equality in care as a benefit of reliable HRII, as it offers everyone access to the same information. However, considering the PHNs’ experiences of patients having problems with HRII and other research that shows low levels of health literacy [22,23], it is probably pertinent to ask how to achieve equitable care. In consultations with Internet-informed patients, promoting equitable care would mean enabling patients with varying predispositions to partake in the general information supply. The PHNs’ described shift in roles and tasks, moving from controlling to coaching increasingly knowledgeable patients, seems to be a step in this direction. Dickerson et al also described how the Internet had changed nurses from educators to knowledge consultants [54]. Previous research has suggested different approaches for coaching patients’ HRII use. Since misconceptions easily arise from inaccurate HRII and poor levels of eHealth literacy, previous studies have emphasized the importance of HCPs in facilitating and encouraging open discussions about HRII [26,39,55]. Active assessment of patients’ use of HRII has been recommended as valuable [13]. Guiding patients to credible websites is also important to ensure that they obtain reliable information and to minimize confusion, anxiety, or poor health decisions based on contradictory information or misunderstood medical jargon [11,31,45]. Research into patients’ views has shown a desire for such navigational help from HCPs [26,56,57]. Since HRII is broad and general, HCPs could play an important role in contextualizing the HRII to the patient’s personal situation [26,53]. It has been suggested that nurses occasionally visit the most popular parenting forums for updates on current health questions, since parents often leave these sites confused by conflicting information [58]. It is also important for HCPs to be aware that even though most patients obtain and are influenced by HRII, far from all of them mention this information to their HCP. Such reluctance may be due to fear of being perceived as challenging, previous bad experiences of presenting HRII, fear of embarrassment, or the perception that the HRII is not important enough [28,39]. Patients with navigational needs have been shown to be less likely to discuss their Internet findings [59], a fact that supports the importance of HCPs actively encouraging discussions about HRII.

### Strengths and Limitations

This study offers both strengths and limitations. The participating PHNs were varied in age and years of professional experience, which strengthens the trustworthiness and transferability of our results [36]. All the participating PHNs were women, which could be considered a limitation. However, most Swedish PHNs are women [60], which should make the results transferable in this context. There is a possibility that PHNs with stronger views on the subject of eHealth were more likely to volunteer for the study, which could represent a bias.

Because they are in accord with previous research, some aspects of our results are also considered transferable to other health care professions. The interviews offered rich data, and saturation was considered reached by the last interviews.

Because four of the study authors are PHNs, like the participants, reflexivity was considered throughout the research process. Familiarity with the attributes of participants can contribute to a better in-depth understanding of the participants’ experiences, but we were alert to avoiding our own projections during the process. In order to consider different perspectives, continuous discussions were held within the research group [61]. All authors were involved in the analytical process, which is considered to strengthen the trustworthiness of the study.

### Implications for Future Research and Practice

PHNs and other HCPs are encouraged to actively raise discussions about patients’ perceptions of HRII, help patients to evaluate and contextualize their Internet findings, and guide patients to credible and evidence-based websites.

This study implies that the phenomenon of Internet-informed patients affects the everyday work and professional role of PHNs. Further research is needed to make a profound analysis of the mechanisms behind ambivalent attitudes, for example, regarding concepts like *power* and *professional identity*, and how attitudes are linked to perceptions of patients’ eHealth literacy levels. Future studies will also explore PHNs’ Internet use and eHealth literacy and how these factors affect attitudes toward Internet-informed patients.

### Conclusions

This study suggests that PHNs are ambivalent about working with Internet-informed patients. While they expressed fundamentally positive attitudes toward increased patient involvement and patient-centered interactions, these attitudes were somewhat overshadowed by reported negative perceptions of the effects and consequences of HRII. Accurate and correctly managed HRII has been shown to offer many possible advantages for patients and health care systems. However, the negative attitudes and apprehensions of HCPs about Internet-informed patients could obstruct the development of increased patient involvement. To promote equitable care in the digital era, increased awareness in health care about useful strategies for overcoming difficulties and embracing benefits of HRII seems to be a legitimate goal.

## Abbreviations

EQUATOR: Enhancing the QUAlity and Transparency of Health Research
HCP: health care professional
HRII: health-related Internet information
ICT: information and communications technology
PHN: primary health care nurse
SFO-V: Strategic Research Area Health Care Science

## References

[1] (2019). World Health Organization.

[2] (2016). Government Offices of Sweden and Swedish Association of Local Authorities and Regions.

[3] (2016). Swedish eHealth Agency.

[4] (2015). Healthcare Guide 1177.

[5] Findahl O (2010). The Swedes and the Internet (Svenskarna och Internet).

[6] TNS Political & Social (2014). European Citizens' Digital Health Literacy.

[7] Fox S (2006). Pew Research Center.

[8] Davidsson P, Thoresson A (2017). The Swedes and the Internet 2017: Survey on Swedes' Internet Habits (Svenskarna och Internet 2017: Undersökning om Svenskarnas Internetvanor).

[9] Clarke MA, Moore JL, Steege LM, Koopman RJ, Belden JL, Canfield SM, Meadows SE, Elliott SG, Kim MS (2016). Health information needs, sources, and barriers of primary care patients to achieve patient-centered care: A literature review. Health Informatics J.

[10] Ramsey I, Corsini N, Peters MDJ, Eckert M (2017). A rapid review of consumer health information needs and preferences. Patient Educ Couns.

[11] McMullan M (2006). Patients using the Internet to obtain health information: How this affects the patient-health professional relationship. Patient Educ Couns.

[12] Townsend A, Leese J, Adam P, McDonald M, Li LC, Kerr S, Backman CL (2015). eHealth, participatory medicine, and ethical care: A focus group study of patients' and health care providers' use of health-related Internet information. J Med Internet Res.

[13] Calvillo J, Román I, Roa LM (2015). How technology is empowering patients? A literature review. Health Expect.

[14] Eysenbach G (2001). What is eHealth?. J Med Internet Res.

[15] Xiang J, Stanley SJ (2017). From online to offline: Exploring the role of eHealth consumption, patient involvement, and patient-centered communication on perceptions of health care quality. Comput Human Behav.

[16] (2000). World Health Organization.

[17] (2018). The Swedish Agency for Health and Care Service Analysis.

[18] (2008). US Department of Health and Human Services.

[19] Hirsch M, Aggarwal S, Barker C, Davis CJ, Duffy JMN (2017). Googling endometriosis: A systematic review of information available on the Internet. Am J Obstet Gynecol.

[20] McNally SL, Donohue MC, Newton KP, Ogletree SP, Conner KK, Ingegneri SE, Kagnoff MF (2012). Can consumers trust Web-based information about celiac disease? Accuracy, comprehensiveness, transparency, and readability of information on the Internet. Interact J Med Res.

[21] Corcelles R, Daigle CR, Talamas HR, Brethauer SA, Schauer PR (2015). Assessment of the quality of Internet information on sleeve gastrectomy. Surg Obes Relat Dis.

[22] Kutner M, Greenburg E, Jin Y, Paulsen C (2006). The Health Literacy of America’s Adults: Results From the 2003 National Assessment of Adult Literacy.

[23] Sørensen K, Pelikan JM, Röthlin F, Ganahl K, Slonska Z, Doyle G, Fullam J, Kondilis B, Agrafiotis D, Uiters E, Falcon M, Mensing M, Tchamov K, van den Broucke S, Brand H, HLS-EU Consortium (2015). Health literacy in Europe: Comparative results of the European health literacy survey (HLS-EU). Eur J Public Health.

[24] Amann J, Rubinelli S, Kreps GL (2015). Revisiting the concept of health literacy: The patient as information seeker and provider. Eur Health Psychol.

[25] Ahmad F, Hudak PL, Bercovitz K, Hollenberg E, Levinson W (2006). Are physicians ready for patients with Internet-based health information?. J Med Internet Res.

[26] El Sherif R, Pluye P, Thoër C, Rodriguez C (2018). Reducing negative outcomes of online consumer health information: Qualitative interpretive study with clinicians, librarians, and consumers. J Med Internet Res.

[27] Norman CD, Skinner HA (2006). eHealth literacy: Essential skills for consumer health in a networked world. J Med Internet Res.

[28] Murray E, Lo B, Pollack L, Donelan K, Catania J, Lee K, Zapert K, Turner R (2003). The impact of health information on the Internet on health care and the physician-patient relationship: National US survey among 1050 US physicians. J Med Internet Res.

[29] Sommerhalder K, Abraham A, Zufferey MC, Barth J, Abel T (2009). Internet information and medical consultations: Experiences from patients' and physicians' perspectives. Patient Educ Couns.

[30] Barnoy S, Volfin-Pruss D, Ehrenfeld M, Kushnir T (2008). Factors affecting nurses' attitudes in Israel toward patients who present them with Internet medical information. Nurs Outlook.

[31] Dilliway G, Maudsley G (2008). Patients bringing information to primary care consultations: A cross-sectional (questionnaire) study of doctors' and nurses' views of its impact. J Eval Clin Pract.

[32] Glenngård AH, Hjalte F, Svensson F, Anell A, Bankauskaite V (2005). Health Systems in Transition: Sweden.

[33] (2008). Swedish Society of Nursing.

[34] Tong A, Sainsbury P, Craig J (2007). Consolidated criteria for reporting qualitative research (COREQ): A 32-item checklist for interviews and focus groups. Int J Qual Health Care.

[35] Graneheim UH, Lundman B (2004). Qualitative content analysis in nursing research: Concepts, procedures and measures to achieve trustworthiness. Nurse Educ Today.

[36] Graneheim UH, Lindgren B, Lundman B (2017). Methodological challenges in qualitative content analysis: A discussion paper. Nurse Educ Today.

[37] World Medical Association (2013). World Medical Association Declaration of Helsinki: Ethical principles for medical research involving human subjects. JAMA.

[38] Öberg U, Isaksson U, Jutterström L, Orre CJ, Hörnsten Å (2018). Perceptions of persons with type 2 diabetes treated in Swedish primary health care: Qualitative study on using eHealth services for self-management support. JMIR Diabetes.

[39] Tan SS, Goonawardene N (2017). Internet health information seeking and the patient-physician relationship: A systematic review. J Med Internet Res.

[40] Hart A, Henwood F, Wyatt S (2004). The role of the Internet in patient-practitioner relationships: Findings from a qualitative research study. J Med Internet Res.

[41] Bowes P, Stevenson F, Ahluwalia S, Murray E (2012). 'I need her to be a doctor': Patients' experiences of presenting health information from the internet in GP consultations. Br J Gen Pract.

[42] Bylund CL, Gueguen JA, Sabee CM, Imes RS, Li Y, Sanford AA (2007). Provider-patient dialogue about Internet health information: An exploration of strategies to improve the provider-patient relationship. Patient Educ Couns.

[43] Dickerson SS, Brennan PF (2002). The Internet as a catalyst for shifting power in provider-patient relationships. Nurs Outlook.

[44] Ahluwalia S, Murray E, Stevenson F, Kerr C, Burns J (2010). 'A heartbeat moment': Qualitative study of GP views of patients bringing health information from the Internet to a consultation. Br J Gen Pract.

[45] Lee K, Hoti K, Hughes JD, Emmerton L (2014). Dr Google and the consumer: A qualitative study exploring the navigational needs and online health information-seeking behaviors of consumers with chronic health conditions. J Med Internet Res.

[46] Schwartz KL, Roe T, Northrup J, Meza J, Seifeldin R, Neale AV (2006). Family medicine patients' use of the Internet for health information: A MetroNet study. J Am Board Fam Med.

[47] White RW, Horvitz E (2010). Web to world: Predicting transitions from self-diagnosis to the pursuit of local medical assistance in Web search. AMIA Annu Symp Proc.

[48] Doherty-Torstrick ER, Walton KE, Fallon BA (2016). Cyberchondria: Parsing health anxiety from online behavior. Psychosomatics.

[49] Te Poel F, Baumgartner SE, Hartmann T, Tanis M (2016). The curious case of cyberchondria: A longitudinal study on the reciprocal relationship between health anxiety and online health information seeking. J Anxiety Disord.

[50] Singh K, Brown RJ (2016). From headache to tumour: An examination of health anxiety, health-related Internet use and 'query escalation'. J Health Psychol.

[51] McManus F, Leung C, Muse K, Williams J (2014). Understanding ‘cyberchondria’: An interpretive phenomenological analysis of the purpose, methods and impact of seeking health information online for those with health anxiety. Cogn Behav Therap.

[52] Ghaddar SF, Valerio MA, Garcia CM, Hansen L (2012). Adolescent health literacy: The importance of credible sources for online health information. J Sch Health.

[53] van der Gugten AC, de Leeuw RJ, Verheij TJ, van der Ent CK, Kars MC (2016). E-health and health care behaviour of parents of young children: A qualitative study. Scand J Prim Health Care.

[54] Dickerson SS, Boehmke M, Ogle C, Brown JK (2005). Out of necessity: Oncology nurses' experiences integrating the Internet into practice. Oncol Nurs Forum.

[55] Gilmour JA, Huntington A, Broadbent R, Strong A, Hawkins M (2012). Nurses' use of online health information in medical wards. J Adv Nurs.

[56] Lee K, Hoti K, Hughes JD, Emmerton L (2017). Dr Google is here to stay but health care professionals are still valued: An analysis of health care consumers' Internet navigation support preferences. J Med Internet Res.

[57] Khoo K, Bolt P, Babl FE, Jury S, Goldman RD (2008). Health information seeking by parents in the Internet age. J Paediatr Child Health.

[58] Porter N, Ispa JM (2013). Mothers' online message board questions about parenting infants and toddlers. J Adv Nurs.

[59] Lee K, Hoti K, Hughes JD, Emmerton LM (2015). Consumer use of "Dr Google": A survey on health information-seeking behaviors and navigational needs. J Med Internet Res.

[60] (2013). Statistics Sweden.

[61] Berger R (2015). Now I see it, now I don’t: Researcher’s position and reflexivity in qualitative research. Qual Res.

